# Typhus Fever

**Published:** 1915-06

**Authors:** 


**Typhus Fever. Previously Shown to be Filterable,** the virus last summer yielded a specific bacillus to Dr. Harry Plotz of Mt. Sinai Hospital, N. Y. A bacterin has been prepared and is to be used in Servia.

